# Making the first moves as a university teacher: Effective teaching of students is a life-long challenge and requires a solid understanding of pedagogical concepts

**DOI:** 10.1038/s44319-025-00600-8

**Published:** 2025-10-27

**Authors:** Ľubomír Tomáška

**Affiliations:** Department of Genetics, Comenius University Bratislava, Faculty of Natural Sciences, Ilkovičova 6, 842 15 Bratislava, Slovak Republic

**Keywords:** Careers, History & Philosophy of Science, Methods & Resources

## Abstract

Many academics are woefully unprepared when they have to start teaching students. This article provides some advice for and personal experience from taking the first steps as a teacher.

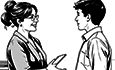

“People in higher education land know the assumption is that your content knowledge expertise is the prerequisite to being a good teacher, and the rest of it just kind of magically happens. Well, it doesn’t.” [Beckie Supiano, 2025]

I begin with a confession: I have no formal education in pedagogy. As highlighted by the quote above, I am not alone. In hindsight, I see this as a significant handicap, especially given that I have been working as a university teacher for more than 30 years.

At the start of my teaching career, I assumed that being a good teacher was simple: just imitate those colleagues I considered good and avoid the styles and behaviors I disliked. However, I quickly encountered a problem—imitation only works within the bounds of one’s own personality and abilities. Beyond that, it will likely turn into a caricature. “Never mind,” I told myself. “I’ll improve by trial and error.” But the issue with this approach is that, however well-intentioned, intuitive attempts at teaching often fail and may even harm students. I eventually realized that many of my mistakes stemmed from ignorance of basic didactic principles. So, I immersed myself in textbooks on teaching and participated in workshops specifically designed for university teachers, where I learned foundational principles of good teaching. I only wish I had done this sooner!

… however well-intentioned, intuitive attempts at teaching often fail and may even harm students.

## The challenge of teaching the first classes

As an excuse, most academics begin teaching during their early PhD years, often as unprepared as I was. In some respects, today’s challenges are even more complex: The average world’s gross enrollment ratio for higher education more than doubled in 23 years—increasing from 19% in 2000 to 43% in 2023 [UNESCO, 2025]—and the students are more diverse in their backgrounds, knowledge, technical and social skills. Educational resources—largely thanks to the internet and social media—are also far more readily available than when I began teaching. In the 1990s, there were no MOOCs (Massive Open Online Courses), educational YouTube videos and animations, podcasts or various online educational platforms. On the one hand, it is wonderful to have a wide range of tools within easy reach; on the other hand, it can be challenging to identify those that are truly relevant and useful.

Furthermore, there is a digital gap between students and teachers as students may be more willing to adopt new tools such as artificial intelligence (AI) than their instructors, even if the latter are young. And many fields have witnessed an explosion of information that overwhelms our ability to fully grasp it. Unfortunately, novice instructors lacking training in pedagogy are not prepared for these challenges. The result is not only a disservice to students but also personal frustration for the teachers. This is a pity, because good teaching can be a source of joy and well-being and has even been shown to enhance research performance [Feldon et al, 2011].

Many universities have initiated pedagogy courses as part of their graduate programs. One example is the course developed by Ed Himelblau at California Polytechnic State University, who recently published an excellent book on the topic [Himelblau, 2024]. I believe such a course should be mandatory for all new instructors before they begin teaching. This article is written for those who may not have that opportunity.

**Figure 1:**
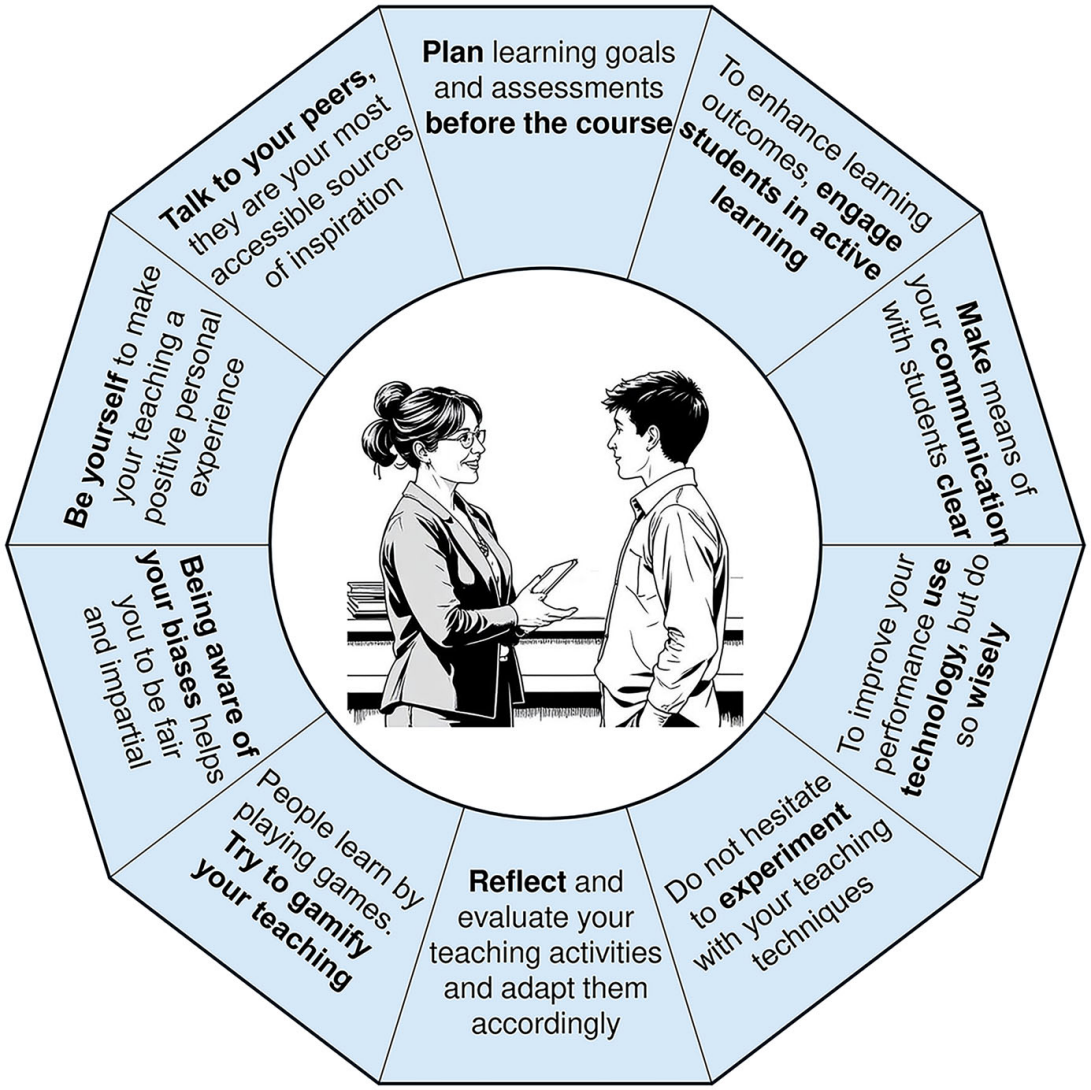
Recommendations for making the first moves as a university teacher are presented as brief annotations. The central illustration, depicting communication between a teacher and a student, was created using the AI Image Generator (https://deepai.org).

Teaching is a complex human endeavor and has been studied extensively, beginning with *Didactica Magna* (*The Great Didactic*) by John Amos Comenius (1592–1670)— arguably the first comprehensive work on pedagogy [Comenius, 1967]—followed by thousands of papers and books, including the most authoritative references [Svinicky et al, 2014; Brinkley et al, 1999; Nilsson, 2016]. Needless to say, there are no simple rules of teaching. To learn how to teach is a career-long run and requires a lot of time and effort. My ambition is to provide a starting block for new “runners” and recommendations that outline key activities involved in university teaching (Fig. 1). While they are drawn from my own experience with teaching students in the life sciences, they are supported by empirical evidence and should also apply to other fields (see Further reading).

To learn how to teach is a career-long run and requires a lot of time and effort.

I am aware that being a university teacher also includes advanced courses for PhD students and activities outside the classrooms, such as supervising students’ research projects. However, to avoid an overly superficial treatment of other responsibilities, I have chosen to focus on directly on formal courses for undergraduates.

Finally, I am certain that many educators might disagree with some items on the list and would propose alternatives. If this article sparks such discussions within university departments, it will have achieved more than its main purpose: to help new teachers in making the first moves in the classrooms.

## Develop a detailed plan before the course begins

Ideally, the teaching-related activities start well ahead of the actual beginning of the class. The sooner you know which course you will teach, the better you can prepare and the less stressful it will be.

The students will expect that you know more about the topic than they can read in a textbook. Therefore, you should aim to master your subject as thoroughly as possible, given the time and opportunities available. Be aware that knowing the essentials about a subject is a part of pedagogical content knowledge, that is, knowledge how to teach a specific topic in a way that makes it understandable. You should think about possible student misconceptions about the subject, how students’ thinking about the topic may change with instruction and identify effective examples, analogies, problems, case studies, visual representations and other strategies to make a specific topic accessible to students and facilitate their learning.

… knowing the essentials about a subject is a part of pedagogical content knowledge, that is, knowledge of how to teach a specific topic in a way that makes it understandable.

When you master the subject, make a list of learning goals. You should start by asking yourself: What should the course accomplish? Consider using a backward design of curriculum development that prioritizes learning outcomes before planning activities and assessments. It starts with identifying what students should know and be able to do by the end of a unit or course and then works backward to design appropriate learning experiences.

When you set the goals of the course, you should elaborate on the means of how to achieve them. To this end, you should address questions such as: What textbook(s) will serve as a reference for the course? How much do the students already know about the subject? What method of teaching—lecture, seminar, group discussion, student-centered or combinations thereof—will I use? How many students will attend the course? The latter question is crucial for choosing teaching approaches that, in the case of small classes, maximize their main benefit of having face-to-face interactions with the students and, for the large courses, minimize the drawbacks associated with lower student engagement, interest, persistence and effective long-term retention of critical-thinking skills (see also Further reading).

How do you assess that the goals of the course were achieved? Assessment is a complex topic that cannot be fully covered here; thus, I just provide some basic terminology and principles. When students think about their assessment, they usually mean an exam designed to measure “how much” they learned in the course or module. Such so-called summative assessments are usually the main basis for a final grade. In addition, you should consider performing an assessment of students’ prior knowledge of a subject, called a diagnostic evaluation. The results can be useful not only for how to approach the subject, but also for students to become aware of their own knowledge level. Finally, you should think of using formative or classroom assessments for obtaining evidence of progress or highlighting areas of difficulty for each student in real time. The book *Classroom Assessment Techniques* is a great resource for various real-time assessments that also stimulate students’ engagement [Angelo and Zakrajsek, 2024]. Ensure that the students are informed about the evaluation criteria at the very beginning of the semester. Present the rules in the first session and post them on the course website for ongoing reference.

## Engage your students in learning by doing

The quality of your teaching can also be evaluated by observing your students during the class. If their main activity is sitting, looking at you or your presentation, and occasionally writing notes, interrupted by daydreaming, there is room for improvement. What matters most is not what you do, but what your students do. Therefore, put emphasis on active learning, which was shown to increase student performance in Science, Technology, Engineering and Mathematics (STEM) [Freeman et al, 2014].

What matters most is not what you do, but what your students do.

Practical courses and seminars are particularly well-suited for student engagement, yet even these formats require careful planning. Students should study the theory behind the techniques in advance, and their preparation should be assessed before they begin their tasks. The students should conduct the experiments themselves rather than merely observing the instructor; your role is to demonstrate the task, then allow them to perform it independently, while you observe, correct mistakes and provide support as needed. Ideally, students will complete the experiment with sensible results, but the primary goal is to practice the technique, interpret the findings and propose follow-up experiments. Seminars are also well-suited for student-centered learning, and active participation should extend beyond the presenting student. You can assign roles such as moderator, opponent or proponent, while your responsibility is to guide the discussion when necessary and step in to resolve any issues that arise.

Active-learning techniques are by no means limited to practicals and seminars but also apply to lecture courses. Just because classical lecturing is the most prevalent form of teaching, it is not automatically the most effective or beneficial approach. For example, “calls to increase the number of students receiving STEM degrees could be answered, at least in part, by abandoning traditional lecturing in favor of active learning” [Freeman et al, 2014]. Therefore, if possible, consider other forms of teaching and discuss with your peers how to try them. Do not be discouraged by the fact that students themselves may appear resistant to active-learning practices, which may be reflected in their evaluation of your performance: a meta-analysis of the student ratings of teachers indicated that they are not related to the learning outcomes [Uttl et al, 2017]. If you have evidence that the intended means will result in better student performance, use them.

You should consider splitting the class into shorter periods in which your monologue is interrupted by intervals during which the students discuss interesting questions and solve problems, individually or in groups, depending on the class size: yes, noise in the classroom can be an indicator of effective teaching. For small- and medium-sized classes, you can try Think-Pair-Share, whereby students first think about a problem, then form a group and discuss their points of view and finally share their opinions [Himelblau, 2024].

Changing the activities has two benefits. First, students’ attention declines over time, and their engagement alternates between attention and nonattention in shorter and shorter cycles as the lecture proceeds, so switching between different actions gives them a chance to reboot. Second, we actually learn by actively doing things either physically or mentally. Nobody learned how to ride a bicycle from watching a cyclist or from a theoretical lecture on keeping the balance on two wheels.

There are other “active learning” activities, some of which can be adopted even for large classes. In addition of using clickers or online quizzing platforms such as Socrative, you can also employ some low-tech approaches. For example, during a lecture students can be asked to write a “one sentence summary” of the major take-home message of the previous part of the lecture, produce a “minute paper” of the discussed principles or pose a question or write a “muddiest point”, that is, identify a concept that is unclear [Angelo and Zakrajsek, 2024].

Although the benefits of students’ engagement are usually appreciated, there are two hurdles that may prevent their active participation in the class. First, it is more difficult and time-consuming to prepare a lesson with well-chosen activities peppered throughout. The second hurdle is that students may sometimes be hesitant to participate in activities that do not follow the standard scenario of the teacher being the messenger and them being receivers. To overcome this barrier and avoid frustrations on both sides, the teacher must explain to students the reason for coming out of their comfort zone and choose the activities such that they discover that this type of class is much more fun and useful than sitting and staring at the board, at the teacher, or outside the window.

One last note: do not confuse engagement with entertainment. While entertainment can be a powerful tool for capturing students’ attention, entertained students may be in a good mood but remain passive. Use entertainment techniques of your choice as gateways that channel students into active learning.

## Make it clear

I have borrowed this dictum from the title of the excellent book by Patrick Winston on communication [Winston, 2020]. In a strict sense, the rule means that you should learn to clearly communicate your thoughts in a lecture or other form of presentation. Although they have their limitations, “lectures can still work” [Svinicky et al, 2014] and it “is likely to survive many more generations” either because of simple economic reasons, or because it has proven to be the best choice for a particular course [Brinkley et al, 1999]. In such instances it is crucial to make it as effective as possible. To this end, Winston’s book, his lecture *How to speak* and a classic antology *Advice to lectures* by Faraday and Bragg [1974] are a great resource for the essential features of an effective presentation.

In the context of this article, the meaning of *Make it clear* is similarly important in a broader sense. Namely, teaching should be free of ambiguities and vagueness. You should make it clear at the beginning of the course what you expect from students and from yourself. For example, it must be clear what the objectives of the course are, how you plan to reach them, how the course will be organized, the basis for students’ evaluation and what course-related activities are expected. You should also provide clear and effective feedback on students’ performance and define the boundaries of your interactions with them. It is important for them to know that you are approachable, but they need to understand the formal requirements of your relationships and when and how they can approach you [Himelblau, 2024].

You should make it clear at the beginning of the course what you expect from students and from yourself.

## Use technologies wisely

The introduction of PowerPoint as part of Windows 3.0 was a major invention that had a great impact on teaching and presenting. It was followed by a wide repertoire of digital tools to share study materials, submit and evaluate assignments, to enable self-assessment or provide feedback. Furthermore, the internet provides access to original publications, videos, animations or MOOCs. Finally, AI tools offer yet another opportunity to enrich teaching and learning.

All these technologies should be considered when preparing the course. However, one must be aware of the fact that inappropriate use can actually compromise teaching [Reich, 2020]. PowerPoint, for instance, is still an essential presentation tool, but it should be used wisely: data visualization pioneer Edward Tufte argues that PowerPoint is inherently presenter-centered. Shifting it toward a content-centered—or, ideally, audience-centered—approach takes deliberate effort, beginning with planning your presentation away from the computer. According to Tufte, starting in PowerPoint too soon often leads to wasted time and a less coherent message. Direct communication is the means of interaction we are best adapted to as a species. Check out Richard Feynman’s physics lectures from the 1960s. I believe that using slides would have diminished their clarity—and their beauty.

One way to avoid this is to prepare the first outlines for your class without slides. First, think about what you want to say and which visual aids would be helpful to convey the message. Would some props help? Can I draw a scheme on a blackboard? Such means can be as effective as slides, with the advantage that they are less likely to split the students’ attention between you and the screen [Winston, 2020].

This is not to say that technology cannot be a helpful companion—quite the contrary. You can show a live microscopic image to illustrate a cellular structure, play an animation or video that recapitulates what you said, wrote or drew. Give students an anonymous online quiz that can be evaluated in real time and show the results. Split students into groups and let them discuss the results of an experiment presented on a slide or in an online publication. Play a part in an online lecture to recapitulate an important concept and underline its importance. There are plenty of useful resources, for example, iBiology platform contains a large number of excellent talks by leading scientists from different fields of biology. Let the students consult an AI tool on a particular problem and discuss the outcome. Find out, which teaching tools are provided by your university and use them. Many universities provide tutorials and organize workshops for teachers on how to use these. Do not miss such opportunities to find a good mixture of technologies facilitating not only better students’ understanding of the topic, but also engagement.

## Do not be afraid to experiment

One neglected feature of teaching that is shared with research is that they are both experimental activities. Just as scientists working at the laboratory bench, teachers can perform experiments aimed at testing particular hypotheses: about communication techniques, about motivational tools, about students and their priorities. When we look at teaching this way, the dichotomy between scientific and pedagogical work is blurred. One should be aware that, as in the lab, experiments in the classroom might fail and jeopardize the aim. To minimize such risk, it is worth to perform an informal pilot experiment with colleagues and subject it to peer evaluation.

Successful experiments with teaching will produce similar emotional satisfaction as when the results of your lab experiment comply with your predictions. Or even better: you observe a serendipitous yet positive outcome.

Experimenting in class can be beneficial also to more experienced teachers. Including a small experiment in the lecture you gave many times prevents making them routines that may become boring for students as well as the teacher. Finally, small shots of adrenaline associated with every good experiment may serve as a relatively potent vaccine against burning out.

## Reflect your teaching experiences

Regardless of your time and effort invested in the preparation, not all parts of the class will go as planned. Keep notes reflecting your experiences, good or bad. Himelblau’s guide provides a template protocol [Himelblau, 2024]. Fill it out immediately after class and address questions such as what went well; what went wrong; which technical issues you encountered; which of your questions stimulated discussion and which were met with silence; what questions were raised by students; and conclude with a list of possible improvements.

In addition to self-evaluation, it is helpful to ask somebody you trust and whose opinion you value to visit your class and give critical feedback on your performance. Instead of a general question, How did I do?, ask him/her specific questions—how frequently could the students perform a task; how these tasks were distributed during the session; did the students receive an immediate feedback; did the students have an opportunity to use higher-order cognitive skills; how much time they were given to think before having to express their opinions; how frequently they worked in groups; whether the assessment of their performance was fair.

In addition to self-evaluation, it is helpful to ask somebody you trust and whose opinion you value to visit your class and give critical feedback on your performance.

Collect all these reflections in a notebook dedicated to the corresponding class. When you encounter any relevant information on the subject you teach, such as an interesting article, animation/video, podcast, make a note. This will make future updates of the course much easier.

## Consider teaching as a game

Childhood is, in large part, filled with playing games as the most natural and effective means of learning. Yet, when children enter formal schooling, these learning tools are systematically stripped away. As I argued earlier [Tomáška, 2022], by the time students reach university, learning often becomes a passive exercise focused on information transfer rather than engagement or exploration. Despite our lifelong affinity for playing—evident in sports and video games—playfulness is still seen as frivolous in academic settings. Yet, games promote motivation, creativity, collaboration and critical thinking. They also improve cognitive and emotional engagement, which is essential for deep learning.

It is important to be aware of potential pitfalls of “gamification”, such as increased logistical demands [Svinicky et al, 2014], as well as heightened anxiety and decreased motivation among students, often resulting from poorly chosen games (Ratino et al, 2023). Therefore, choose the game wisely and you will make your course more engaging, dynamic and effective [Tomáška, 2022].

## Be aware of your own biases

Individuals can differ greatly in how they evaluate the quality of students’ responses, either due to variability or “noise” in their judgments [Kahneman et al, 2021], or due to biases. You should become aware of these limitations and try to minimize their effects.

The imperative to be aware of one’s biases does not apply only to teachers, but to anyone involved in evaluating the performance of others. For example, as referees of the Horizon Europe program, we were advised to “…consider that without realizing it, you might make quick choices based on prior experiences, assumptions and interpretations.” During the short briefing, we were supposed to watch a short video *Understanding unconscious bias*, by the Royal Society, with the following recommendations: deliberately slow down decision-making; reconsider reasons for decisions; question cultural stereotypes; and monitor each other for unconscious bias. The final sentence of the presentation constitutes the essence of what applies not only to proposal evaluation, but also to teaching: “We cannot cure unconscious bias, but with self-awareness, we can address it.”

You should also learn about proven strategies that are effective in enhancing classroom equity: giving students opportunities to think and talk about the subject; encouraging, demanding and actively managing the participation of all students; building an inclusive and fair classroom community; and monitoring behavior to cultivate divergent thinking.

## Be (the best of) yourself

In evolutionary developmental biology, the term phenotypic plasticity refers to the ability of a single genotype to develop different phenotypes in response to environmental conditions. Similarly, teachers’ personal traits affect the effectiveness of their teaching, as well as the well-being, retention and engagement of their students. You should take this into account and try your best to reach your personal limits. It was shown that affective factors—such as student interest, self-efficacy and mindset—play a crucial role in supporting students’ persistence and retention in STEM. Instructors can strongly influence the development of these factors. Likewise, prior studies have shown that instructor immediacy—students’ perceptions of closeness to and trust in their instructor—is an important contributor to success in biology courses. Even a simple skill, such as calling on students by name, increases the likelihood of instructor immediacy. Inclusion of a personal story can have a great positive effect, as a narrative related to the subject can be not only a very strong attention attractor, but also offer increased comprehension, interest and engagement.

… teachers’ personal traits affect the effectiveness of their teaching, as well as the well-being, retention and engagement of their students. You should take this into account and try your best to reach your personal limits.

You do not need to limit your conversations with students to the topic of the session. Even the non-content language can foster rapport, reduce threat and build community. Naturally, the choice of the techniques to achieve this goal is dependent on your personality. For example, if you know that you can make people laugh, use this skill in your teaching. If, in contrast, you feel uneasy about telling funny stories, do not push it. The personality of every individual is an exclusive blend of characteristics, and you should take advantage of those that will make you a memorable teacher with a unique style that facilitates students’ learning.

Empirical data show that teachers’ pedagogical performance is not primarily influenced by technology, curriculum reforms or accreditation standards, but by other teachers [Reich, 2020]. Just as peer-learning is an effective tool for teaching students, it is a powerful means of teacher education. Thus, identify a community of your peers who are willing to share their teaching experience with you. If there is no platform enabling their periodic interactions in your department, be active and establish it. For example, declare one coffee-break during the week or month as teachers’ discussion club meeting. Talk about your successes and failures, present case studies of what worked or did not work, discuss how you tried to resolve a problematic issue and ask for suggestions for how to do it better, compare the contents of your courses and identify things that can be improved. Such discussion clubs will be beneficial not only for you to become a better teacher, but also for your department, fostering a culture of continuous improvement in education.

## Conclusions

You should keep in mind that a good teacher is a guide, not a medium, and this requires a lot of effort, time and courage. To put it simply, the art of teaching is not given, it must be earned. The positive message is that your hard work will be richly rewarded—not necessarily through a larger bank balance, but through the growth in knowledge of the young people you have had the privilege to guide through university. As the astronomer and teacher Christa McAuliffe put it: “I touch the future. I teach.”

… your hard work will be richly rewarded—not necessarily through a larger bank balance, but through the growth in knowledge of the young people you have had the privilege to guide through university.

However, when you reach a level that you become satisfied with, be aware of the danger of falling into stereotypes. The world is changing more rapidly than ever, and this affects not only the ever-expanding knowledge in your field, but also students’ expectations, skills and behavior. Furthermore, the effects—good or bad–of AI on education are difficult, if not impossible, to predict. To be prepared for these changes means to have an open mind and the ability and willingness to adapt. Noam Chomsky's witty comment on teaching: “If you’re teaching today what you were teaching 5 years ago, either the field is dead or you are,” should be complemented by “If you’re teaching today the same way you were teaching 5 years ago, either you are dead or the world stopped changing, which is far less likely.”

## Supplementary information

Peer Review File
