# Supplementary material for: Making the first moves as a university teacher: Effective teaching of students is a life-long challenge and requires a solid understanding of pedagogical concepts
Source: EMBO Rep. 2025 Oct 27;26(22):5365–71. doi: 10.1038/s44319-025-00600-8 (PMC12635050; doi:10.1038/s44319-025-00600-8)
Supplement: Supplementary file 1 — Peer Review File [file 44319_2025_600_MOESM1_ESM.pdf]

EMBO reports

Making the first moves as a university teacher

Lubomir Tomaska

Corresponding author(s): Lubomir Tomaska (lubomir.tomaska@uniba.sk)

Review Timeline:

Submission Date:	16th Sep 25
Editorial Decision:	23rd Sep 25
Revision Received:	25th Sep 25
Accepted:	7th Oct 25

Editor: Holger Breithaupt

Transaction Report: This article was editorially reviewed.
